# *Euphorbia bicolor* (*Euphorbiaceae*) Latex Extract Reduces Inflammatory Cytokines and Oxidative Stress in a Rat Model of Orofacial Pain

**DOI:** 10.1155/2019/8594375

**Published:** 2019-09-12

**Authors:** Paramita Basu, Rebecca S. Hornung, Dayna L. Averitt, Camelia Maier

**Affiliations:** Department of Biology, Texas Woman's University, Denton, 76204 TX, USA

## Abstract

Recent studies have reported that the transient receptor potential V1 ion channel (TRPV1), a pain generator on sensory neurons, is activated and potentiated by NADPH oxidase-generated reactive oxygen species (ROS). ROS are increased by advanced oxidation protein products (AOPPs), which activate NADPH oxidase by upregulating Nox4 expression. Our previous studies reported that *Euphorbia bicolor* (*Euphorbiaceae*) latex extract induced peripheral analgesia, partly *via* TRPV1, in hindpaw-inflamed male and female rats. The present study reports that *E*. *bicolor* latex extract also can evoke analgesia *via* reduction of oxidative stress biomarkers and proinflammatory cytokines/chemokines in a rat model of orofacial pain. Male and female rats were injected with complete Freund's adjuvant (CFA) into the left vibrissal pad to induce orofacial inflammation, and mechanical allodynia was measured by the von Frey method. Twenty-four hours later, rats received one injection of *E. bicolor* latex extract or vehicle into the inflamed vibrissal pad. Mechanical sensitivity was reassessed at 1, 6, 24, and/or 72 hours. Trigeminal ganglia and trunk blood were collected at each time point. In the trigeminal ganglia, ROS were quantified using 2′,7′-dichlorodihydrofluorescein diacetate dye, Nox4 protein was quantified by Western blots, and cytokines/chemokines were quantified using a cytokine array. AOPPs were quantified in trunk blood using a spectrophotometric assay. *E. bicolor* latex extract significantly reduced orofacial mechanical allodynia in male and female rats at 24 and 72 hours, respectively. ROS, Nox4, and proinflammatory cytokines/chemokines were significantly reduced in the trigeminal ganglia, and plasma AOPP was significantly reduced in the trunk blood of extract-treated compared to vehicle-treated rats. *In vitro* assays indicate that *E*. *bicolor* latex extract possessed antioxidant activities by scavenging free radicals. Together our data indicate that the phytochemicals in *E. bicolor* latex may serve as novel therapeutics for treating oxidative stress-induced pain conditions.

## 1. Introduction

Pain is a major submodality of the somatosensory system that serves as a warning to alert the organism to actual injury or the threat of injury. However, pain also can develop in the absence of injury or continue following the resolution of injury leading to a transition from acute to chronic pain. Acute and chronic pain manifest as the development and maintenance of hyperalgesia and/or allodynia. The International Association for the Study of Pain defines hyperalgesia as an increased sensitivity to noxious stimuli, while allodynia is defined as an increased sensitivity to nonnoxious stimuli. The transition mechanism from acute to chronic pain is not entirely understood, and once chronic pain has developed, it is hard to treat without the long-term use of addictive opioid-based narcotics. The identification of nonopioid pharmaceutical targets is needed to improve chronic pain management.

A potential target for chronic pain management may be managing the noxious effects of oxidative stress on peripheral sensory neurons. Patients with spinal cord injury and diabetic neuropathy [1, 2] suffer from hyperalgesia and allodynia arising, in part, from oxidative stress due to either excessive formation of reactive oxygen species (ROS) or a decrease in antioxidant capacity [3]. This is supported by preclinical studies reporting that superoxide (reactive oxygen) and peroxynitrite (nitrogen species) led to the development of hyperalgesia [4–6]. Furthermore, ROS are key mediators in the development of peripheral and central sensitization in various pain etiologies, including neuropathic, inflammatory, opioid-induced, and orofacial pain [7]. One of the most common orofacial pain disorders is temporomandibular joint disorder (TMD) pain. It has been reported recently that oxidative stress contributes to TMD pain. Oxidative stress biomarkers are significantly elevated in TMD patients [8] correlating with a corresponding reduction in total antioxidant capacity [9]. These data indicate that pain medications that include antioxidant and free radical scavenging activity may be beneficial for reducing TMD pain.

Recent studies have reported that ROS may cause pain through activation of the transient receptor potential V1 ion channel (TRPV1), a pain generator in peripheral sensory neurons. It was reported that ROS, such as nitric oxide, can activate TRPV1 [10, 11] to increase entry of calcium ions in the cytosol, thus enhancing the channel's sensitivity to acid and heat and contributing to pain signalling [10]. Also, TRPV1 can be activated and potentiated by NADPH oxidase generated ROS [12, 13]. The source of *in vivo* ROS is attributed to advanced oxidation protein products (AOPPs), dityrosine-containing cross-linking protein products formed primarily because of oxidative stress [14]. AOPPs are known to activate NADPH oxidase by increasing the expression of its regulatory subunits, Nox1, Nox2, and Nox4 [13, 15]. Nox4 mRNA is present in sensory neurons, and Nox4-derived ROS contribute to pain signalling after peripheral nerve injury [16]. Indeed, AOPPs activate TRPV1 *via* NADPH oxidase 4-dependent ROS production leading to the development of hyperalgesia [13].

The development of hyperalgesia and allodynia also involves sensitization of sensory neurons by inflammatory cytokines and chemokines. Tissue injury induces the release of bradykinin, which leads to the release of tumor necrosis factor alpha (TNF*α*), interleukin 6 (IL-6), and interleukin 1 beta (IL-1*β*) [17]. Cytokines directly sensitize sensory neurons by increasing neuronal responses to thermal, mechanical, and chemical stimuli [18–21]. This can occur *via* TRPV1 as TNF*α* enhances capsaicin responses in sensory neurons through neuronal production of prostaglandins [18]. Also, TNF*α*, IL-1*β*, and IL-6 increase neuronal excitability *via* TRPV1 [22, 23]. Furthermore, the TNF*α* receptors are coexpressed with TRPV1 in sensory neurons and IL-1*β* and IL-6 increase TRPV1 expression in sensory neurons *via* the extracellular signal-regulated kinase pathway [24, 25].

Many *Euphorbia* species are known to possess phytochemicals with antioxidant and anti-inflammatory properties [26–33]. We previously reported that *Euphorbia bicolor* (*Euphorbiaceae*) latex extract evokes long-lasting peripheral analgesia that does not involve opioid receptors but occurs in part through TRPV1, in a rat inflammatory pain model [34]. Given that other *Euphorbia* species display antioxidant and anti-inflammatory properties and *E. bicolor* is able to induce robust analgesia, we hypothesized that *E. bicolor* latex extract evokes analgesia *via* downregulation of oxidative stress biomarkers and proinflammatory cytokines in a rat model of orofacial pain.

## 2. Materials and Methods

### 2.1. Plant Collection and Latex Extract Preparation

*E. bicolor* (*Euphorbiaceae*) plants were collected from prairies in Denton County, Texas, USA, and a voucher specimen was placed in the Texas Woman's University Herbarium. Fresh latex was collected from the stem, leaf, and inflorescence bracts and extracted in 80% methanol (1 : 40 *w*/*v*) at room temperature for two days. The extract was centrifuged at 3500 rpm for 20 minutes, and the supernatant was filtered through Whatman #54 filter paper and stored at -20°C for future use.

### 2.2. Phytochemical Analyses

#### 2.2.1. Total Phenolic Content

Total phenolic content of the latex extract was determined by the Folin–Ciocalteu method [35]. 400 *μ*L of latex extract was mixed with 1.6 mL of 7.5% sodium carbonate and 2 mL of Folin–Ciocalteu reagent (diluted 10 times in deionized water). The reaction mixtures were incubated at room temperature for 1 hour. The absorbances were measured at 765 nm. A gallic acid standard curve was used to estimate the total phenolic content in the latex extract, and the results were expressed as mg gallic acid equivalents (GAE)/g of latex fresh weight (FW).

#### 2.2.2. Total Flavonoid Content

Total flavonoid content was determined by the method of Ordonez *et al*. [36]. 500 *μ*L of latex extract was mixed with 500 *μ*L of 2% aluminium chloride prepared in ethanol and incubated at room temperature for 1 hour. The absorbances were measured at 430 nm. A quercetin standard curve was used to estimate the total flavonoid content in the latex extract, and the results were expressed as mg quercetin equivalents (QE)/g of latex FW.

#### 2.2.3. Total Proanthocyanidin Content

Total proanthocyanidin content was determined by the method of Aiyegoro and Okoh [37]. 500 *μ*L of latex extract, 3 mL of 4% (*v*/*v*) vanillin-methanol, and 1.5 mL of hydrochloric acid were mixed and vortexed thoroughly and allowed to stand at room temperature for 15 minutes. The absorbances were measured at 500 nm. The total proanthocyanidin content of the latex extract was estimated using a gallic acid standard curve and expressed as mg GAE/g of latex FW.

#### 2.2.4. Total Terpenoid Content

Total terpenoid content was estimated by the modified method of Ghorai *et al*. [38]. The latex extract was centrifuged at 4000 g for 15 minutes at room temperature. 200 *μ*L of supernatant was mixed with 1.5 mL of chloroform. The standard was prepared by adding 200 *μ*L of 2000 *μ*g/mL of linalool in methanol to 1.5 mL of chloroform, and serial dilutions were prepared in the range of 100 mg/200 *μ*L-1 mg/200 *μ*L. The sample and standard mixtures were vortexed thoroughly and allowed to stand for three minutes, after which 100 *μ*L of sulfuric acid (H_2_SO_4_) was added. The sample mixture was incubated at room temperature for 1.5-2 hours in the dark, and the standard was incubated for 5 minutes at room temperature in the dark. A reddish-brown precipitate formed at the end of the incubation. The supernatants were decanted from both sample and standard mixtures, and the precipitates were dissolved in 1.5 mL of 95% (*v*/*v*) methanol and vortexed thoroughly until dissolved completely. The absorbances were measured at 538 nm using 95% (*v*/*v*) methanol as a blank. The total terpenoid concentration of the latex extract was estimated as linalool equivalents using a linalool standard curve.

### 2.3. *In Vitro* Antioxidant and Radical Scavenging Activities of *E. bicolor* Latex Extract

A series of *in vitro* assays were employed to determine the *in vitro* antioxidant and radical scavenging activities of *E. bicolor* latex extract. All assays were performed according to the original methods specified for each assay and modified by Basu and Maier [39].

#### 2.3.1. Ferric Reducing Power

Ferric reducing power of the latex extract was performed by the method of Oyaizu [40]. Increasing concentrations (20 *μ*g/mL–100 *μ*g/mL) of 1 mL latex extract were mixed with 2.5 mL of 0.2 M phosphate buffer (pH 6.6) and 2.5 mL of 1% *w*/*v* K_3_Fe(CN)_6_. The reaction mixtures were incubated at 50°C for 20 min, after which 2.5 mL of 10% *w*/*v* trichloroacetic acid (TCA) was added and the mixtures were centrifuged at 3000 rpm for 10 min. 2.5 mL of supernatants was mixed with 2.5 mL deionized water and 0.5 mL of 0.1% *w*/*v* FeCl_3_. The results were presented as absorbances measured at 700 nm. Butylated hydroxylated toluene (BHT) was used as a positive control.

#### 2.3.2. 2,2′-Azino-bis-(3-ethylbenzothiazoline-6-sulphonic Acid) (ABTS) Scavenging Activity

2,2′-Azino-bis-(3-ethylbenzothiazoline-6-sulphonic acid) (ABTS) scavenging activity was determined by the method of Re *et al*. [41]. ABTS working solution was prepared by mixing equal amounts of 7 mM ABTS and 2.4 mM potassium persulfate in the dark at room temperature for 12 h. Increasing concentrations (20 *μ*g/mL-100 *μ*g/mL) of 1 mL latex extract were mixed with 1 mL of ABTS solution. The reaction mixtures were incubated at room temperature for 7 minutes, and absorbances were measured at 734 nm. The results were presented as percent inhibition of ABTS radical calculated according to equation (1). Ascorbic acid was used as a standard.

(1) $$\begin{array}{c} \%\text{ of free radical scavenging activity}=\left(\frac{\left[{\text{Abs}}_{\text{control}}-{\text{Abs}}_{\text{sample}}\right]}{{\text{Abs}}_{\text{control}}}\right)\times 100. \end{array}$$

#### 2.3.3. 2,2-Diphenyl-1-picrylhydrazyl (DPPH) Scavenging Activity

Free radical scavenging activity was determined by measuring the 2,2-diphenyl-1-picrylhydrazyl (DPPH) radical scavenging activity was determined using a modified method of Gülçin *et al*. [42]. Increasing concentrations (20 *μ*g/mL–100 *μ*g/mL) of 0.05 mL latex extract were mixed with 2.95 mL of 0.1 mM DPPH in methanol, thoroughly vortexed, and incubated in the dark for 30 minutes. The absorbances were recorded at 517 nm, and results were presented as percent inhibition of DPPH radical calculated according to equation (1). Ascorbic acid was used as a standard.

#### 2.3.4. Hydrogen Peroxide Scavenging Activity

Hydrogen peroxide scavenging activity was determined by the method of Ruch *et al*. [43]. Increasing concentrations (20 *μ*g/mL-100 *μ*g/mL) of 4 mL latex extract were mixed with 600 *μ*L of 4 mM H_2_O_2_ solution prepared in 0.1 M phosphate buffer (pH 7.4). The reaction mixtures were incubated for 4 minutes, and the absorbances were recorded at 230 nm against blank solution containing latex extract without H_2_O_2_. The results were presented as percent inhibition of H_2_O_2_ radical calculated according to equation (1). Butylated hydroxylated toluene (BHT) was used as a standard.

#### 2.3.5. Nitric Oxide Scavenging Activity

Nitric oxide (NO) scavenging activity was determined by the modified method of Balakrishnan *et al*. [44]. Increasing concentrations (20 *μ*g/mL-100 *μ*g/mL) of 1 mL latex extract were mixed with 2 mL of sodium nitroprusside prepared in phosphate-buffered saline (PBS). The reaction mixtures were incubated at 25°C for 150 min, after which 0.5 mL of Griess reagent was added to the incubating mixture. The absorbances were measured at 540 nm, and results were presented as percent inhibition of NO radical calculated according to equation (1). Quercetin was used as a standard.

### 2.4. IC_50_ and Pearson's Correlation

IC_50_ values, i.e., the concentration required to scavenge 50% of free radicals, were calculated by linear regression analysis. Pearson's correlations were performed between the IC_50_ values of radical scavenging activities and total phenolic, flavonoid, proanthocyanidin, and terpenoid contents of the latex extract.

### 2.5. Animals

A total of 70 male and 70 female (250-350 g) adult Sprague-Dawley rats (Charles River Laboratories, Wilmington, MA, USA) were separated by sex and housed in cages in a 12 : 12 hour light : dark cycle with *ad libitum* access to food and water. Rats were maintained in the animal facility for a minimum of five days before testing. All studies were conducted under the approval of the Texas Woman's University Institutional Animal Care & Use Committee and under the strict guidelines of Committee for Research and Ethical Issues of the International Association for the Study of Pain and Animal Welfare Act, implementing Animal Welfare Regulations, and the principles of the *Guide for the Care and Use of Laboratory Animals*. The experimenters were blind to the treatment groups during behaviour testing, and all rats were acclimated to the testing room and apparatus twenty-four hours prior to testing.

### 2.6. Behaviour Testing

Sensitivity to a nonnoxious mechanical (touch) stimulus was measured as the force to withdraw from contact with a blunt von Frey filament (North Coast Medical Inc., Gilroy, CA), as previously characterized [45]. For this test, a filament of 2.0 g for noninflamed tissue and 0.16 g for inflamed tissues was first applied to the vibrissal pad. When no response was observed, 30 seconds later, the next thicker filament was applied and the process was repeated until a withdraw response was observed. When a withdraw response was observed, the next thinnest filament was applied 30 seconds later, and the process was repeated until no withdraw response was observed. The filament size that produced at least three withdrawals was recorded as the threshold grams of pressure required to elicit a head withdrawal response as a measure of mechanical allodynia.

For the experiment, baseline mechanical sensitivity was recorded followed by injections of 50 *μ*L complete Freund's adjuvant (CFA; 1 : 1 in 0.9% sterile saline; Sigma-Aldrich, St. Louis, MO, USA) into the left vibrissal pad to induce inflammation. Mechanical allodynia was then confirmed 24 hours following CFA injections. Rats received one injection of either *E. bicolor* latex extract (300 *μ*g/mg in 0.9% saline and <5% methanol; *n* = 10 males and *n* = 10 females) or vehicle (0.9% saline and <5% methanol; *n* = 10 males and *n* = 10 females) into the inflamed vibrissal pad. Mechanical allodynia was then reassessed at 1, 6, and 24 hours in males and 1, 6, and 72 hours in females. The latex extract concentration and time points were selected based on our previous report that the onset of extract-induced analgesia in male and female rats with hindpaw inflammation occurs at 6 and 72 hours, respectively [34].

### 2.7. Tissue Collection and Analysis

A total of 60 male and 60 female rats (*n* = 10 per time point, per sex, and per treatment) were injected with CFA into the left vibrissal pad and saline into the right vibrissal pad. At 1, 6, and 24 hours in males and 1, 6, and 72 hours in females, rats were rapidly decapitated under brief gas anesthesia (isoflurane; 3%). A separate control group of rats (*n* = 10 males and *n* = 10 females) received only one saline injection into the left vibrissal pad. 24 hours post saline injection, male and female rats were rapidly decapitated under brief gas anesthesia (isoflurane; 3%). Trunk blood was collected in BD Vacutainer® spray-coated K2EDTA collection tubes on ice (Pulmolab, CA, US). Immediately after collection, the cells were removed from plasma by centrifugation at 1000–2000 x g for 10 minutes. The separated plasma was then transferred into clean polypropylene tubes and stored at −80°C. The trigeminal ganglia (TGs) were bilaterally extracted from the same rats and either immediately used or stored at −80°C.

#### 2.7.1. Quantification of Advanced Oxidation Protein Product (AOPP)

The level of AOPP was quantified in the plasma of male and female rats treated with either *E. bicolor* latex extract or vehicle (*n* = 10 per sex and per treatment) by the modified method of Witko-Sarsat *et al*. [14]. For this method, 2 mL of plasma was diluted in PBS at a 1 : 5 ratio; 10 *μ*L of 1.16 M potassium iodide (KI) was added, followed by 20 *μ*L of acetic acid after 2 min. The absorbance of the reaction mixture was measured at 340 nm against a blank containing 2 mL of PBS, 10 *μ*L of KI, and 20 *μ*L of acetic acid. The AOPP level was determined as *μ*mol·L^−1^ chloramine-T equivalents by using a chloramine-T linear curve ranging from 0 to 100 *μ*M.

#### 2.7.2. Quantification of Reactive Oxygen Species (ROS)

The level of ROS was detected in the TGs isolated from male and female rats treated with either *E. bicolor* latex extract or vehicle (*n* = 4 per sex and per treatment) by the method of Chung *et al*. [46], using a cell-permeant 2′,7′-dichlorodihydrofluorescein diacetate (H_2_DCFDA), a reduced form of fluorescein used as ROS indicator. The nonfluorescent H_2_DCFDA is converted into the highly fluorescent 2′,7′-dichlorofluorescein when the acetate groups of H_2_DCFDA are cleaved by intracellular oxidation. H_2_DCFDA detects levels of hydrogen peroxide, peroxyl radicals, and peroxynitrite in dissociated cells and intact tissues [47]. Freshly extracted TGs were quickly washed, minced, and incubated in 96-well plates with 200 *μ*L PBS for 30 minutes at 37°C. After 30 minutes, the background fluorescence was detected (Biotek's Synergy HT fluorimeter) at 485 nm excitation and 535 nm emission. After recording the background reading, H_2_DCFDA was added into each well at a final concentration of 10 *μ*M. The plates were incubated for another 30 minutes at 37°C, after which the fluorescence was remeasured. The level of ROS was detected as the intensity of fluorescence after subtracting the background fluorescence. TGs from the saline-treated vibrissal pad were used as internal controls and data normalization.

#### 2.7.3. Quantification of Nox4 Protein Expression by Western Immunoblotting

TGs isolated from male and female rats treated with either *E. bicolor* latex extract or vehicle (*n* = 4 per sex and per treatment) were homogenized in radioimmunoprecipitation (RIPA) lysis and extraction buffer containing Halt protease inhibitor cocktail (Thermo Scientific; part no. 78430) to prevent proteolysis. Tissue homogenization was performed at 6.5 m/s, 3 times for 10 seconds each, in a FastPrep®-24 homogenizer (MP Biomedicals; Santa Ana, CA). Homogenates were centrifuged at 13000 rpm for 15 minutes at 4°C. The protein concentration was determined by using Pierce BCA™ protein assay kit. Protein lysate (30 *μ*g) was resolved by sodium dodecyl sulfate polyacrylamide gel electrophoresis (SDS-PAGE), followed by transfer onto a polyvinylidene difluoride (PVDF) membrane (Bio-Rad; Hercules, CA) using Bio-Rad's Western blot unit. Membranes were blocked with 5% bovine serum albumin (BSA) in TBS-Tween 20 and probed with monoclonal anti-Nox4 (1 : 500; 67 kDa; Abcam) overnight, followed by incubation with secondary antibody (1 : 8000, Alexa Fluor) at room temperature for one hour. To confirm the selectivity of the antibody, blots were preincubated with blocking peptide (ab155071, Abcam) for Nox4. Immunoreactive bands were detected with a Licor Odyssey imaging system. Beta actin was used as an internal control to normalize protein expression, and the percentage of *β*-actin was calculated based on at least four independent experiments.

#### 2.7.4. Cytokine and Chemokine Proteome Profiling

A Proteome Profiler Rat Cytokine Array Kit, column A (R&D Systems Inc., Minneapolis, MN), containing 4 membranes coated with 29 cytokines/chemokines (Table 1) was probed with rat protein samples extracted from TGs, and the relative cytokine levels were compared. Briefly, 200 *μ*g of protein extracted from vehicle- or extract-treated TGs from male or female rats (*n* = 4 males and *n* = 4 females per treatment) was mixed with a cocktail of biotinylated detection antibodies. The nitrocellulose membranes were blocked according to the manufacturer's protocol. The protein and antibody mixtures were incubated with the membrane containing immobilized antibodies for 29 rat cytokines. Bound protein was detected with streptavidin conjugated to horseradish peroxidase (HRP). Membranes were washed and developed with chemiluminescent detection reagents. The cytokine/chemokine spots were detected with a Bio-Rad ChemiDoc™ MP imaging system.

### 2.8. Statistical Analyses

All data were analyzed using GraphPad Prism software version 7 (GraphPad, San Diego, CA, USA). Behavioural data were presented as means ± SEM grams and analyzed by repeated measures two-way analysis of variance (ANOVA) followed by Bonferroni post hoc analysis. *In vitro* antioxidant and radical scavenging activities were analyzed by ordinary two-way ANOVA followed by Bonferroni post hoc analysis with statistical significance tested at *p* ≤ 0.05. AOPP and ROS levels in saline- and CFA-treated animals were analyzed by the Student *t*-test with a statistical significance tested at *p* ≤ 0.05. Western blot bands were quantified by densitometry using the Licor Odyssey imaging system Image Studio 2.0, and cytokine/chemokine levels were quantified by densitometry using ImageJ version 1.8.0 (National Institutes of Health, Bethesda, MD, USA). Densitometry data were analyzed by two-way ANOVA followed by Bonferroni post hoc analysis with statistical significance tested at *p* ≤ 0.05.

## 3. Results

### 3.1. *E*. *bicolor* Latex Extract Displays *In Vitro* Antioxidant and Free Radical Scavenging Activities

**Figure 1:**
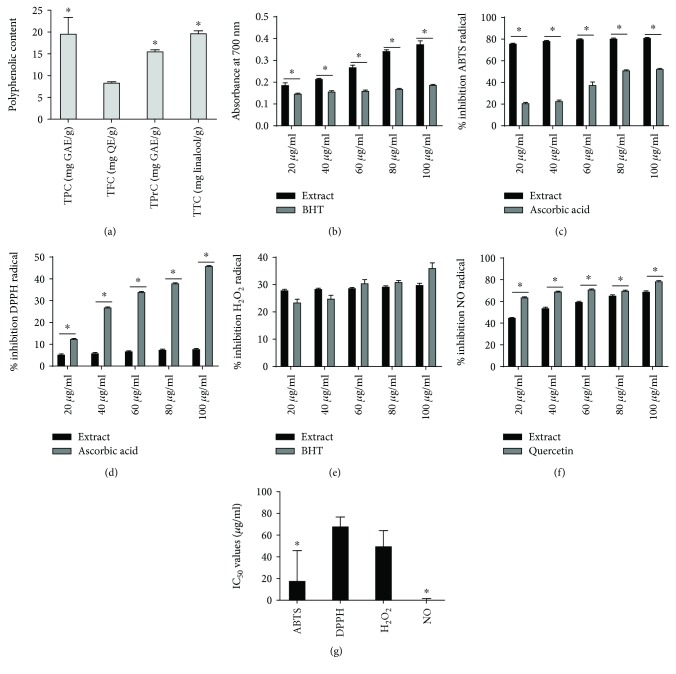
Total polyphenolic contents, *in vitro* antioxidant, *in vitro* free radical scavenging activities, and IC_50_ values of *E*. *bicolor* latex extract. Total phenolic (TPC), flavonoid (TFC), proanthocyanidin (TPrC), and terpenoid (TTC) contents of latex extract (a). Asterisks indicate significance compared to TFC at *p* ≤ 0.05 by one-way ANOVA with Tukey's analysis. Reducing power (b), percent inhibition of the 2,2′-azino-bis-(3-ethylbenzothiazoline-6-sulphonic acid) (ABTS) radical (c), percent inhibition of the 2,2-diphenyl-1-picrylhydrazyl (DPPH) radical (d), percent inhibition of the hydrogen peroxide (H_2_O_2_) radical (e), and percent inhibition of the nitric oxide (NO) radical (f). For each assay, extract (black bars) was compared to its corresponding standard (grey bars) by two-way ANOVA with Bonferroni post hoc analysis at *p* ≤ 0.05. ^∗^Significant difference compared to the positive control. IC_50_ values of radical scavenging activities of latex extract (g). Asterisks indicate significance compared to IC_50_ of DPPH scavenging activity at *p* ≤ 0.05 by one-way ANOVA with Tukey's analysis.

*E*. *bicolor* latex extract significantly contained more total phenolics (TPC), proanthocyanidins (TPrC), and terpenoids (TTC) than total flavonoids (TFC) (*F* (3, 8) = 24.34; *p* ≤ 0.05) (Figure 1(a)). The *in vitro* antioxidant and radical scavenging activities of the *E*. *bicolor* latex extract were evaluated through a series of *in vitro* assays. *E*. *bicolor* latex extract significantly and concentration-dependently increased the ferric reducing activity compared to the standard BHT (*F* (1, 20) = 902.7; *p* ≤ 0.05) (Figure 1(b)). The extract inhibited approximately 80% of the ABTS radical at 20-100 *μ*g/mL, which was a significantly greater inhibition as compared to that of the ascorbic acid standard (*F* (1, 20) = 6729; *p* ≤ 0.05) (Figure 1(c)). Additionally, the latex extract reduced approximately 8% of DPPH radical at 20-100 *μ*g/mL (Figure 1(d)), whereas H_2_O_2_ radical was reduced by approximately 30% at 20-100 *μ*g/mL (Figure 1(e)). The ascorbic acid standard induced significantly higher scavenging activities than the latex extract on DPPH (*F* (1, 20) = 100787; *p* > 0.05) (Figure 1(d)), while the extract induced H_2_O_2_ scavenging activities comparable to the BHT standard (*F* (1, 20) = 0.34; *p* > 0.05) (Figure 1(e)). The latex extract reduced nitric oxide (NO) radical in a concentration-dependent manner (Figure 1(f)) and was significantly higher compared to the quercetin standard (*F* (1, 20) = 2983; *p* > 0.05).

Total polyphenolic contents were estimated and correlated with the IC_50_ values of the radical scavenging activities of the *E. bicolor* latex extract (Figure 1(g) and Table 2). Latex extract significantly (*F* (3, 8) = 10.71; *p* ≤ 0.05) quenched 50% of ABTS, DPPH, H_2_O_2_, and NO radicals (Figure 1(g)). The IC_50_ values of the latex extract required to quench 50% of free radicals are ranked as follows: NO (1.6 ± 0.04 *μ*g/mL), ABTS (18.2 ± 13 *μ*g/mL), H_2_O_2_(50 ± 6.6 *μ*g/mL) < DPPH (68.4 ± 3.8 *μ*g/mL). ABTS radical scavenging activity showed high correlations with flavonoid (*R*^2^ = 0.866) and proanthocyanidin (*R*^2^ = 0.861) contents of the latex extract. The DPPH (*R*^2^ = 0.929) and NO (*R*^2^ = 0.975) radical scavenging activities of the extract showed high correlations with phenolics (*R*^2^ = 0.929) and terpenoids (*R*^2^ = 0.979). H_2_O_2_ radical scavenging activity showed low to negative correlations with polyphenolics, indicating that other phytochemicals are involved. NO radical scavenging activity showed positive and high correlations with phenolics (*R*^2^ = 0.975) and terpenoids (*R*^2^ = 0.999) (Table 2).

### 3.2. *E*. *bicolor* Latex Extract Reduced Orofacial Mechanical Allodynia

**Figure 2:**
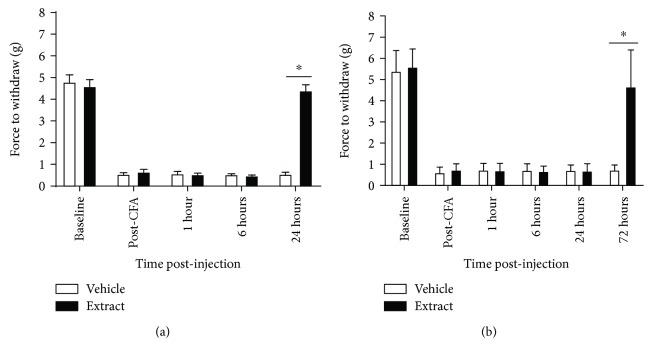
*E*. *bicolor* latex extract reduces orofacial mechanical sensitivity in both male and female rats. Orofacial mechanical allodynia was reduced in males (a) at 24 hours and in females (b) at 72 hours post *E*. *bicolor* latex extract injection (closed bars) as compared to vehicle treatment (open bars). ^∗^*p* ≤ 0.05 compared to vehicle by repeated measures two-way ANOVA with Bonferroni post hoc analysis.

*E*. *bicolor* latex extract significantly reduced orofacial mechanical sensitivity in male rats (*F* (1, 18) = 38.6; *p* ≤ 0.05) at 24 hours (Figure 2(a)) and in female rats (*F* (1, 18) = 15.71; *p* ≤ 0.05) at 72 hours (Figure 2(b)) as compared to vehicle-treated rats and in accordance with our previous timeline of analgesia in hindpaw-inflamed rats [34]. In male rats, extract treatment significantly increased the withdrawal threshold from 0.6 g to 4.4 g, and in female rats, extract treatment significantly increased the withdrawal threshold from 0.74 g to 4.7 g. No significant differences were observed between the vehicle- and extract-treated groups at 1 and 6 hours in male and at 1, 6, and 24 hours in female rats (*p* > 0.05).

### 3.3. *E*. *bicolor* Latex Extract Reduced AOPP, ROS, and Nox4 Levels

**Figure 3:**
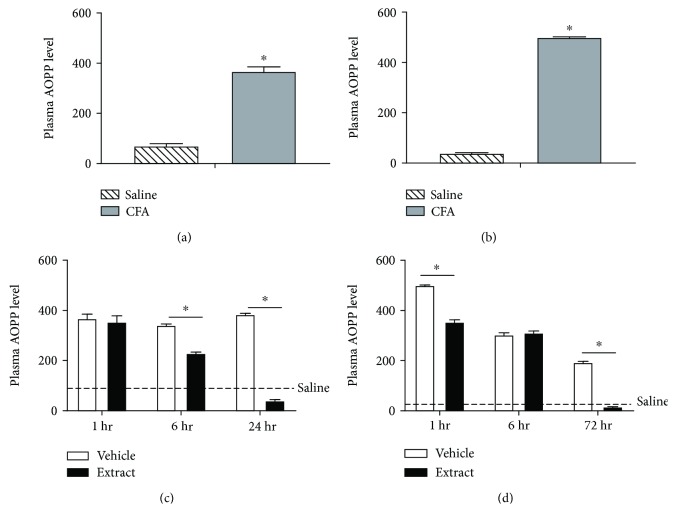
*E*. *bicolor* latex extract reduces plasma AOPP levels in both male and female rats. CFA inflammation (closed bars) increased AOPP levels in male (a) and female (b) rats as compared to saline treatment (diagonal bars). ^∗^*p* ≤ 0.05 compared to vehicle by the Student *t*-test analysis. Plasma AOPP levels decreased in males (c) at 6 and 24 hours and in females (d) at 1 and 72 hours post *E*. *bicolor* latex extract injection (closed bars) as compared to vehicle treatment (open bars). Dotted lines indicate the plasma AOPP level in noninflamed rats. ^∗^*p* ≤ 0.05 compared to vehicle by two-way ANOVA with Bonferroni post hoc analysis.

**Figure 4:**
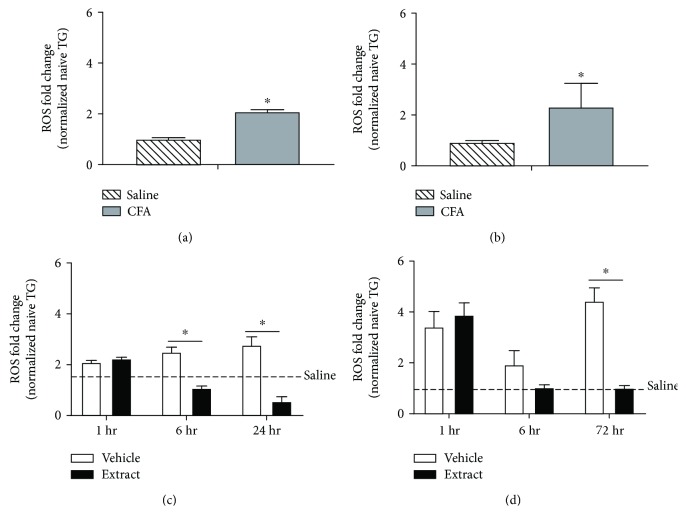
*E*. *bicolor* latex extract reduces ROS levels in both male and female rats. CFA inflammation (closed bar) increased ROS levels in the trigeminal ganglia of male (a) and female (b) rats as compared to saline treatment (diagonal bar). ^∗^*p* ≤ 0.05 compared to vehicle by the Student *t*-test analysis. ROS levels decreased in the trigeminal ganglia of males (c) at 6 and 24 hours and females (d) at 72 hours post *E*. *bicolor* latex extract injection (closed bars) as compared to vehicle treatment (open bars). Dotted lines indicate the ROS level in noninflamed rats. ^∗^*p* ≤ 0.05 compared to vehicle by two-way ANOVA with Bonferroni post hoc analysis.

**Figure 5:**
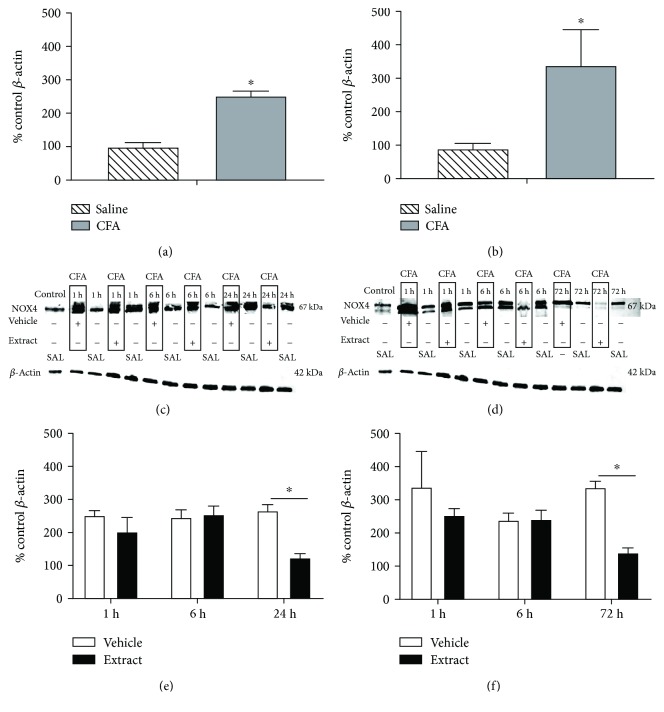
*E*. *bicolor* latex extract reduces Nox4 protein expression in both male and female rats. CFA inflammation (gray bars) increased Nox4 protein expression in the trigeminal ganglia of male (a) and female (b) rats as compared to saline treatment (diagonal bars). ^∗^*p* ≤ 0.05 compared to vehicle by the Student *t*-test analysis. *E*. *bicolor* latex extract induced significant reduction in Nox4 protein expression in male rats ((c) representative immunoblot) at 24 hours posttreatment ((e) quantification of immunoblots) and in female rats ((d) representative immunoblot) at 72 hours posttreatment ((f) quantification of immunoblots). Note that the 1-hour CFA-treated band is 25 hours following CFA and 1 hour following vehicle treatment and the control band is 25 hours following saline. ^∗^*p* ≤ 0.05 compared to vehicle by two-way ANOVA with Bonferroni post hoc analysis (c, d).

CFA inflammation significantly increased plasma AOPP levels in both male (*t* = 14.66, df = 18; *p* ≤ 0.05) (Figure 3(a)) and female (*t* = 135.5, df = 18; *p* ≤ 0.05) (Figure 3(b)) rats as compared to vehicle-treated controls. Female rats had significantly greater CFA-evoked AOPP levels when compared to males (*F* (1, 36) = 24.18; *p* ≤ 0.05). *E. bicolor* extract treatment significantly decreased the AOPP levels in males at 6 and 24 hours (*F* (1, 54) = 212.8; *p* ≤ 0.05) (Figure 3(c)) and at 1 and 72 hours posttreatment in female rats (*F* (1, 54) = 395.5; *p* ≤ 0.05) (Figure 3(d)). CFA inflammation also significantly increased ROS levels in the trigeminal ganglia of both male (*t* = 11.75, df = 6) (Figure 4(a)) and female (*t* = 4.06, df = 6) (Figure 4(b)) rats compared to vehicle-treated controls, and CFA-evoked ROS were comparable between the sexes (*F* (1, 12) = 0.02; *p* > 0.05). Extract treatment significantly decreased ROS levels in males at 6 and 24 hours (*F* (1, 18) = 63.43; *p* ≤ 0.05) (Figure 4(c)) and at 72 hours posttreatment in female rats (*F* (1, 18) = 12.36; *p* ≤ 0.05) (Figure 4(d)). Further, CFA inflammation significantly increased Nox4 protein expression in the trigeminal ganglia of both male (*t* = 15.99, df = 6) (Figure 5(a)) and female (*t* = 4.62, df = 6) (Figure 5(b)) rats compared to vehicle-treated controls. No significant difference was observed in CFA-evoked Nox4 protein expression between male and female rats (*F* (1, 12) = 1.96; *p* > 0.05). Extract treatment significantly decreased Nox4 expression at 24 hours posttreatment in male rats (*F* (1, 18) = 36.24; *p* ≤ 0.05) (Figure 5(e)) and at 72 hours posttreatment in females (*F* (1, 18) = 23.03; *p* ≤ 0.05) (Figure 5(f)).

### 3.4. *E*. *bicolor* Latex Extract Modulated Cytokines/Chemokines

**Figure 6:**
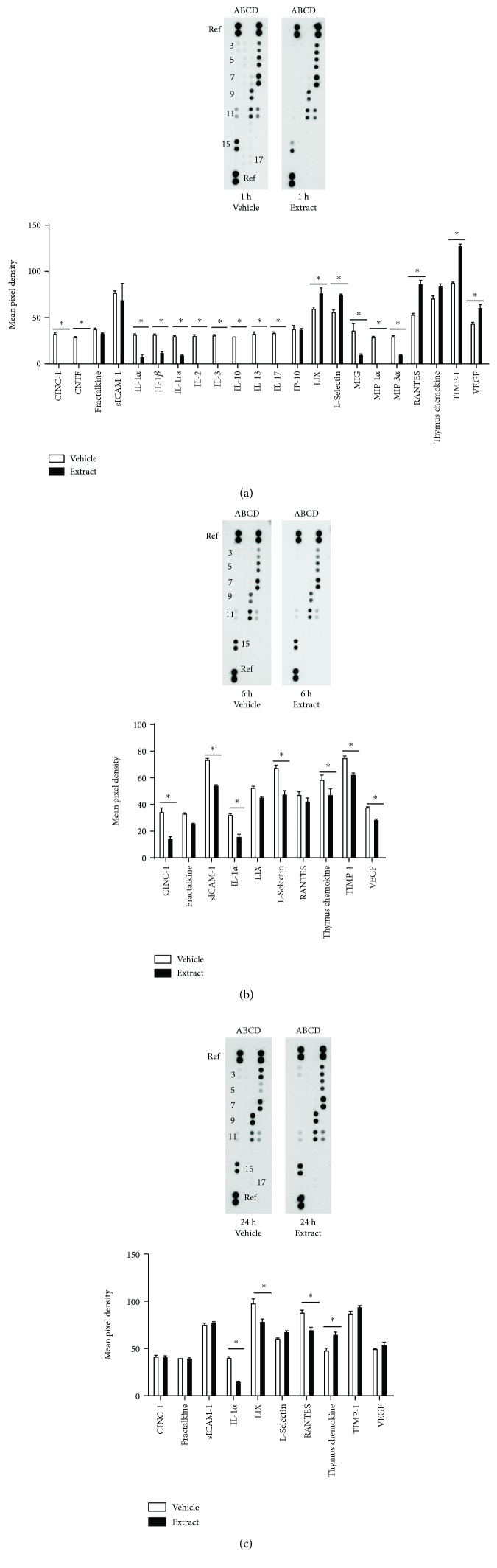
*E*. *bicolor* latex extract modulated cytokines/chemokines in the trigeminal ganglia of male rats. Representative cytokine/chemokine membranes and the respective mean pixel density of detected cytokine or chemokine from the array from extract- (closed bars) and vehicle-treated (open bars) male rats at 1 hour (a), 6 hours (b), and 24 hours posttreatment (c). Columns A–D illustrate cytokine or chemokine expression levels by row (see Table 1). ^∗^*p* ≤ 0.05 compared to vehicle by two-way ANOVA with Bonferroni post hoc analysis.

In male rats, *E*. *bicolor* latex extract significantly downregulated several proinflammatory cytokines (notably IL-1*α*, IL-1*β*, IL-2, IL-3, and IL-17), chemotactic cytokines (MIP-1*α* and MIP-3*α*), and proinflammatory chemokines (CINC-1, MIG) and upregulated an anti-inflammatory chemokine (TIMP) in the trigeminal ganglia 1 hour post *E. bicolor* latex extract treatment compared to vehicle controls (*F* (1, 44) = 125.2; *p* ≤ 0.05) (Figure 6(a)). At 6 hours of treatment, proinflammatory cytokines (sICAM-1, IL-1*α*) and proinflammatory chemokines (CINC-1, VEGF) were significantly downregulated compared to vehicle controls (*F* (1, 20) = 256.7; *p* ≤ 0.05) (Figure 6(b)). At 24 hours post *E. bicolor* latex extract treatment, the proinflammatory cytokine IL-1*α* and proinflammatory chemokines LIX and RANTES were significantly downregulated compared to vehicle controls (*F* (1, 20) = 12.64; *p* ≤ 0.05) (Figure 6(c)).

**Figure 7:**
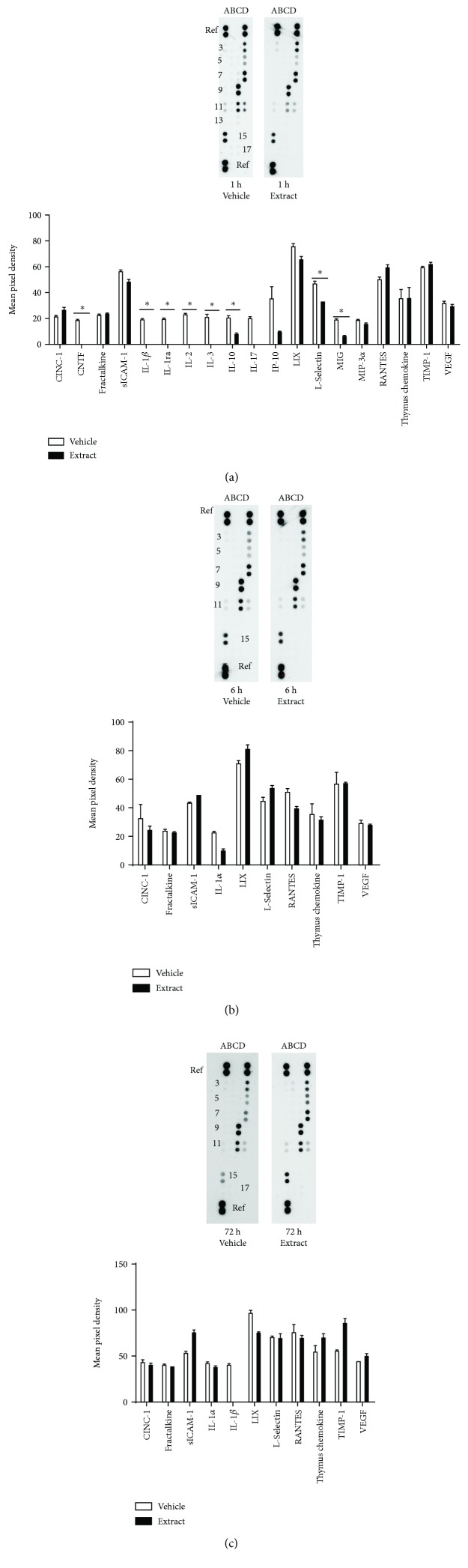
*E*. *bicolor* latex extract modulated cytokines/chemokines in the trigeminal ganglia of female rats. Representative cytokine/chemokine membranes and the respective mean pixel density of detected cytokine or chemokine in the array from extract- (closed bars) and vehicle-treated (open bars) female rats 1 hour (a), 6 hours (b), and 72 hours posttreatment (c). Columns A–D illustrate cytokine or chemokine expression levels by row (see Table 1). ^∗^*p* ≤ 0.05 compared to vehicle by two-way ANOVA with Bonferroni post hoc analysis.

In female rats, *E*. *bicolor* latex extract significantly downregulated several proinflammatory cytokines (notably IL-1*β*, IL-2, and IL-3) and proinflammatory chemokines (MIG) in the trigeminal ganglia 1 hour post *E. bicolor* latex extract treatment compared to vehicle controls (*F* (1, 38) = 189.5; *p* ≤ 0.05) (Figure 7(a)). No significant differences between vehicle- and extract-treated groups were observed at 6 hours (*F* (1, 20) = 0.85; *p* > 0.05) (Figure 7(b)) or 72 hours. At 72 hours, a reduction in the proinflammatory cytokine IL-1*β* was observed and an increase in the anti-inflammatory chemokine TIMP was observed.

## 4. Discussion

Pain and inflammatory conditions are treated mainly with opioids and nonsteroidal anti-inflammatory drugs (NSAIDs), which are responsible for adverse reactions, such as gastrointestinal disturbances, renal damage, respiratory depression, and possible dependence [48–50]. These negative side effects have led to the search for alternative therapeutics, such as phytomedicines [51–54]. As extracts from different species of *Euphorbia* possess antioxidant and anti-inflammatory activities [55–57], we have examined the previously untested *E. bicolor* as a potential phytomedicine. Here, we report for the first time that *E*. *bicolor* latex extract (1) possesses *in vitro* antioxidant and free radical scavenging activities; (2) reduces orofacial mechanical allodynia in both male and female rats; (3) downregulates the oxidative stress biomarkers AOPP, ROS, and Nox4; and (4) alters the expression levels of pro- and anti-inflammatory cytokines/chemokines.

*E*. *bicolor* latex extract displayed *in vitro* free radical scavenging activities by inhibiting ABTS, DPPH, H_2_O_2_, and NO. The ferric reducing power of the latex extract was significantly more potent than that of the BHT standard. The DPPH radical scavenging activity of the latex extract was found to be significantly lower than ABTS scavenging activity. This could be explained by the fact that the latex extract contains more hydrophobic than hydrophilic phytochemicals with antioxidant properties, since the ABTS assay is more suitable for hydrophilic antioxidants while the DPPH assay is more applicable for hydrophobic antioxidants [58]. In addition, total phenolic and terpenoid contents of the latex extract resulted in stronger DPPH and NO scavenging activities, whereas total flavonoid content contributed to ABTS scavenging activity. Low and negative correlation values indicate that other groups of phytochemicals present in the latex extract contributed to the ABTS and H_2_O_2_ radical scavenging activities. Therefore, the differences in the correlations between IC_50_ values and polyphenolic contents explain why DPPH, H_2_O_2_, and NO radical scavenging activities of the extract are not as potent as their corresponding standards.

The orofacial region is innervated by the trigeminal nerves and is one of the most densely innervated regions of the body making trigeminal system-associated pain, such as migraine, headache, temporomandibular joint disorder, and trigeminal neuralgia, difficult to manage. In the present study, *E*. *bicolor* latex extract significantly reduced orofacial mechanical sensitivity in both male and female rats at 24 hours and 72 hours, respectively, which correspond to the same onset time of analgesia observed in our previous studies on hindpaw-inflamed male and female rats [34]. In support, a recent systematic review reported that extracts from various plants are quite effective at alleviating orofacial pain in preclinical experimental models [59]. For example, plant extracts from *Sida cordifolia* [60], *Hyptis pectinata* [61, 62], *Hyptis fruticosa* [63], *Ocimum basilicum* [64], *Acmella oleracea* [65], and *Syzygium cumini* [66] reduce orofacial pain. This suggests that plant phytochemicals may be an effective alternative or adjuvant therapy to opioids and NSAIDs for managing orofacial pain.

Elevated AOPP levels are linked to many diseases, such as cancer [67], chronic irritable bowel syndrome [68], coronary artery disease [69], and diabetes [70, 71], and are thought to contribute to pain arising from these diseases. In the present study, CFA-evoked inflammation significantly increased plasma AOPP levels, corroborating a previous study reporting that CFA induced a 1.6-fold increase in AOPP levels in rats [13]. Interestingly, we also found that CFA evoked higher AOPP levels in female compared to male rats, indicating that females may have a higher level of oxidative stress in response to CFA. Saline treatment induced a decrease in the level of AOPP in female rats. This could be attributed to the antioxidant properties of estrogens. Several studies reported the neuroprotective effect of estradiol through the involvement of antioxidant signalling [72–75]. Estradiol also upregulated the expression of antioxidant enzyme manganese superoxide dismutase (MnSOD) *via* activation of the MAP kinase signalling pathway, which further confirms the antioxidant properties of estrogens [76]. Therefore, it can be speculated that the endogenous estradiol reduced the level of plasma AOPP in saline-treated female rats.

Treatment with *E*. *bicolor* latex extract reduced plasma AOPP levels in male rats at 6 and 24 hours and in female rats at 72 hours. Together, these data indicate that the antioxidant activity of *E*. *bicolor* latex extract correlates to the onset of analgesia but varies by tissue type and sex. Composite methanol extract of *Aegle marmelos*, *Azadirachta indica*, *Murraya koenigii*, *Ocimum sanctum* leaves, and *Syzygium cumini* fruits [77] and ethyl acetate extract of *Anthyllis henoniana* flowers [78] also reduce AOPP levels in alloxan-induced diabetic rats. The aforementioned studies reported the effects of extracts on the AOPP level in male rats only, whereas the present study reports the effects of *E*. *bicolor* latex extract on the AOPP levels in both male and female rats. To the best of our knowledge, this is the first study that reports the sex differences in AOPP level.

Generated ROS as a result of nerve injury or inflammation in peripheral tissues are known to induce pain behaviours [79–81]. In our study, ROS levels were significantly and comparably increased in the trigeminal ganglia of CFA-inflamed male and female rats. These results are in accordance with those of a study reporting that CFA inflammation in rat masseter muscle significantly upregulated ROS levels in the trigeminal ganglia as compared to naïve male rats [46]. Niu *et al*. [82] also reported the significant upregulation of ROS in CFA-induced mechanical muscular hyperalgesia in male rats. The present study reports that *E*. *bicolor* latex extract significantly reduced CFA-evoked ROS levels in the trigeminal ganglia of both male and female rats and along the same time course as that for the AOPP level reduction. Other *Euphorbia* species also display antioxidant activity by reducing ROS levels. For example, *Ricinus communis* reduces ROS generation in macrophage cells [83] and *E*. *supina* reduces ROS generation in fibrosarcoma cells [84]. The production of ROS is a physiological function of the nicotinamide adenine dinucleotide phosphate (NADPH) oxidase, a membrane-bound enzyme complex [85]. In rodents, four different catalytic subunits of NOX genes (NOX1-NOX4) are expressed in a tissue-specific manner [86]. Nox4 has been known to contribute to pain signalling after peripheral nerve injury [16]. In the present study, CFA inflammation significantly and comparably upregulated Nox4 protein expression in both males and females and *E. bicolor* latex extract treatment decreased Nox4 expression in both male and female rats at 24 hours and 72 hours, respectively. The time point for decreased Nox4 protein expression corresponded to that of analgesia onset in both male and female rats, indicating that the extract-induced analgesia may be working at least in part by downregulating Nox4 protein. Since it has been reported that ROS can activate TRPV1 [10, 11] and that *E*. *bicolor* evokes analgesia in part *via* TRPV1 [34], it is possible that *E. bicolor* latex extract treatment reduces AOPP, Nox4, and ROS levels leading to reduced TRPV1 activity contributing to analgesia. Thus, *E. bicolor* latex phytochemicals can reduce oxidative stress effects on TRPV1, thus reducing pain.

The present study focused on the effects of *E*. *bicolor* latex extract on AOPP-mediated activation of TRPV1 *via* Nox4-dependent production of ROS. Other studies [11, 87, 88] have also reported the involvement of Nox1-derived ROS in dorsal root ganglion neurons and Schwann cells under different pain conditions. Marone *et al*. reported that stimulation of proalgesic transient receptor potential ankyrin 1 (TRPA1) channel led to the activation of NOX1/2 in the soma of trigeminal ganglion neurons [89]. The TRPA1 activation and ensuing oxidative stress may sensitize the meningeal nociceptors and the second order trigeminal neurons to show periorbital allodynia, which might be relevant to glyceryl trinitrate-evoked migraine-like headaches in humans. Savini *et al*. found that capsaicin regulates Ecto-NOX1 (plasma membrane external NADH oxidase) expression through TRPV1 and ROS in human platelets [90]. Therefore, future studies will explore the potential reduction of Nox1-mediated ROS production by *E*. *bicolor* latex extract in different pain conditions.

ROS are also known to modulate proinflammatory cytokines [91–93]. In the current study, several proinflammatory cytokines/chemokines were detected in CFA-treated trigeminal ganglia, similar to reports from a rat model of temporomandibular joint disorder pain [94]. The anti-inflammatory activity of the extract was displayed by not only inhibiting proinflammatory cytokines/chemokines but also increasing the expressions of anti-inflammatory cytokines/chemokines. In the present study, significantly upregulated cytokines/chemokines were found, such as ICAM-1, CINC-3, IL-3, thymus chemokine, GM-CSF, VEGF, fractalkine, MIP-3*α*, TNF-*α*, CINC-2*α*/*β*, CNTF, IL-17, IL-1*β*, IL-13, TIMP-1, LIX, L-selectin, IFN-*γ*, and RANTES. Durham *et al*. showed that several cytokines/chemokines, including LIX, L-selectin, TIMP-1, and VEGF, were significantly upregulated in male rats at 2 hours post-TNF*α* injection [95]. Similar results were obtained in the present study at 1 hour post extract injection. Inflammation and pronociceptive cytokines, such as TNF*α*, modulate the expression of other proinflammatory cytokines within the trigeminal ganglion, thus promoting sensitization of neurons [95]. In the present study, 1 hour post extract treatment in males significantly reduced several proinflammatory cytokines, including IL-1*β* and IL-17, which are known to directly activate nociceptors [96, 97]. While this reduction did not yet translate into pain inhibition, further reduction of the proinflammatory cytokines, such as IL-1*α*, at 6 and 24 hours postinjection correlated to a reduction in pain behaviour in male rats.

Chemokines, such as monokine induced by interferon-*γ* (MIG), macrophage inflammatory protein 1*α* (MIP-1*α*), and MIP-3*α*, play an important role in rheumatoid arthritis pathology [98–102]. In the present study, all the above-mentioned chemokines were significantly downregulated in male rats. Other cytokines implicated in rheumatoid arthritis pathology, such as serum levels of soluble intercellular adhesion molecule-1 (sICAM-1) and vascular endothelial growth factor (VEGF), were significantly reduced in male rats at 6 hours post extract treatment. Also, a chemokine named RANTES (or CCL5) that is involved in T cell migration and immunity during infection was significantly reduced in male rats at 24 hours [103–105]. No significant differences in cytokine/chemokine levels were observed between vehicle- and extract-treated groups at 6 hours. *E. bicolor* latex extract significantly increased tissue inhibitors of metalloproteinase (TIMP) level in the trigeminal ganglia of female rats at 72 hours. Others have reported that intrathecal injection of TIMPs significantly reduces neuropathic pain [106] and inhibits joint damage by osteoarthritis in rats [107]. Together, these data indicate that *E*. *bicolor* latex phytochemicals may be effective against neuropathic pain and osteoarthritis.

The present study also reports a sexually dimorphic modulation of cytokines/chemokines by *E*. *bicolor* latex treatment. In female rats, fewer cytokines/chemokines, such as CNTF, lL-1*β*, IL-1ra, IL-2, IL-3, IL-10, L-selectin, and MIG, were significantly altered at 1 hour posttreatment. While lL-1*β* was no longer detectable in males after 1 hour, it was present and lowered by *E. bicolor* treatment in females at 72 hours. During acute inflammatory responses, resident leukocytes present in the resting tissues modulate the levels of circulating cytokines and recruit blood leukocytes. Scotland *et al*. reported that the numbers of leukocytes present in the naïve peritoneal and pleural cavities were higher in female than in male rodents, indicating that increased leukocyte population in female rats may contribute to the recognition and elimination of infectious stimuli without recruiting neutrophils or producing excessive cytokines [108]. The results on sex differences in cytokine/chemokine levels reported in the current study support previously reported differences in tissue and immune cell phenotype between males and females.

## 5. Conclusions

**Figure 8:**
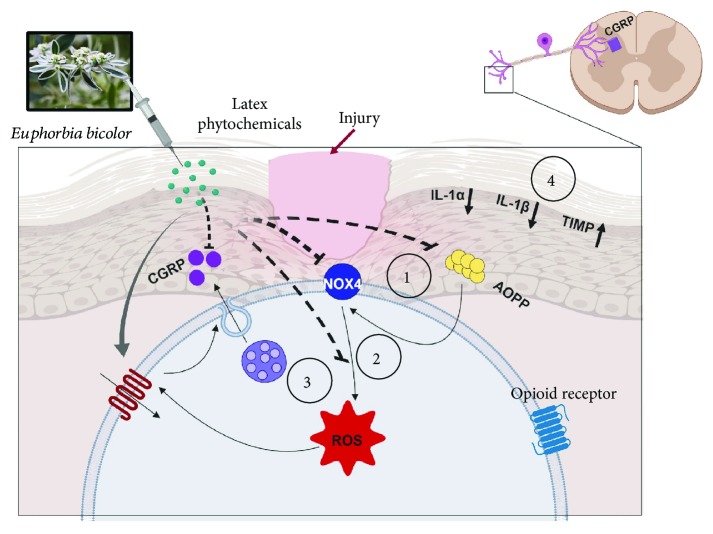
Proposed model of the mechanisms involved in *E*. *bicolor* latex extract-evoked peripheral, nonopioid analgesia. Local injection of *E*. *bicolor* latex phytochemicals at the site of injury reduces plasma advanced oxidation protein product (AOPP) levels (1), which leads to decreased expression of Nox4 protein (2) and reduced levels of reactive oxygen species (ROS) (3). This reduction in ROS leads to a reduction in TRPV1 activity and the release of the proinflammatory peptide calcitonin gene-related peptide (CGRP) in conditions where oxidative stress triggers pain. Latex phytochemicals also reduce proinflammatory and increase anti-inflammatory cytokines/chemokines in a sexually dimorphic manner, which may underlie sex differences in the onset of *E. bicolor*-evoked analgesia (4). This model was created with BioRender http://BioRender.com.

ROS are key players in the development of peripheral and central sensitization in various pain etiologies, including neuropathic, inflammatory, opioid-induced, and orofacial pain. We provide evidence for multiple nonopioid mechanisms that contribute to peripheral analgesia induced by *E*. *bicolor* latex extract (Figure 8). We propose that local injection of *E. bicolor* latex phytochemicals at the site of an injury significantly reduces oxidative stress by reducing AOPP and ROS levels and Nox4 protein expression in parallel with reduced orofacial mechanical allodynia in both male and female rats. Our previous study reported that *E*. *bicolor* latex extract induced long-lasting peripheral analgesia in both male and female rat model of inflammatory pain without involving the opioid receptors [34]. Together, these data indicate that *E. bicolor* latex phytochemicals may be effective at alleviating acute or chronic pain that is driven by nociceptor activation, oxidative stress, and/or inflammation.

## Figures and Tables

**Table 1:** Rat cytokine/chemokine array coordinates (adapted from the manufacturer's guidelines for the Proteome Profiler Rat Cytokine Array Kit, column A, R&D Systems).

	A	B	C	D
1	Ref spot	—	—	Ref spot
2	Ref spot	—	—	Ref spot
3	CINC-1	IL-1*α* or **IL-1F1**	IL-13	RANTES or **CCL5**
4	CINC-1	IL-1*α* or **IL-1F1**	IL-13	RANTES or **CCL5**
5	CINC-2*α*/*β*	IL-1*β* or **IL-1F2**	IL-17	Thymus chemokine or **CXCL7**
6	CINC-2*α*/*β*	IL-1*β* or **IL-1F2**	IL-17	Thymus chemokine or **CXCL7**
7	CINC-3	IL-1ra or **IL-1F3**	IP-10 or **CXCL10**	TIMP-1
8	CINC-3	IL-1ra or **IL-1F3**	IP-10 or **CXCL10**	TIMP-1
9	CNTF	IL-2	LIX	TNF-*α* or **TNFSF1A**
10	CNTF	IL-2	LIX	TNF-*α* or **TNFSF1A**
11	Fractalkine or **CX3CL1**	IL-3	L-Selectin or **CD62L/LECAM-1**	VEGF or **VEGF-A/vasculotropin**
12	Fractalkine or **CX3CL1**	IL-3	L-Selectin or **CD62L/LECAM-1**	VEGF or **VEGF-A/vasculotropin**
13	GM-CSF	IL-4	MIG or **CXCL9**	—
14	GM-CSF	IL-4	MIG or **CXCL9**	—
15	sICAM-1/**CD54**	IL-6	MIP-1*α* or **CCL3**	—
16	sICAM-1/**CD54**	IL-6	MIP-1*α* or **CCL3**	—
17	IFN-*γ*	IL-10	MIP-3*α* or **CCL20**	Negative control or control (-)
18	IFN-*γ*	IL-10	MIP-1*α* or **CCL20**	Negative control or control (-)
19	Ref spot	—	—	—
20	Ref spot	—	—	—

Alternate nomenclatures are in bold.

**Table 2:** Correlation between IC_50_ of *in vitro* radical scavenging activities and phenolic, flavonoid, proanthocyanidin, and terpenoid contents of *E. bicolor* latex extract.

	Correlation (*R*)			
	TPC	TFC	TPrC	TTC
ABTS radical	0.500	0.866	0.861	0.640
DPPH radical	0.929^∗^	-0.370	0.361	0.979^∗^
H_2_O_2_ radical	-1.00	-0.002	0.011	-0.985^∗^
NO radical	0.975^∗^	0.223	-0.214	0.999^∗^

TPC: total phenolic content; TFC: total flavonoid content; TPrC: total proanthocyanidin content; TTC: total terpenoid content. The coefficient values with asterisks indicate significant difference at *p* ≤ 0.05.

## Data Availability

The data used to support the findings of this study are included within the article.
